# Dairy intake in relation to metabolic health status in overweight and obese adolescents

**DOI:** 10.1038/s41598-022-22827-4

**Published:** 2022-11-01

**Authors:** Shahnaz Amani Tirani, Saeideh Mirzaei, Ali Asadi, Masoumeh Akhlaghi, Parvane Saneei

**Affiliations:** 1 Department of Community Nutrition, School of Nutrition and Food Science, Nutrition and Food Security Research Center, Students’ Research Committee, Isfahan University of Medical Sciences, Isfahan, Iran; 2 Department of Community Nutrition, School of Nutrition and Food Science, Shiraz University of Medical Sciences, Shiraz, Iran; 3 Department of Exercise Physiology, School of Physical Education and Sport Sciences, University of Tehran, Tehran, Iran; 4 Department of Community Nutrition, School of Nutrition and Food Science, Nutrition and Food Security Research Center, Isfahan University of Medical Sciences, PO Box 81745-151, Isfahan, Iran

**Keywords:** Nutrition, Public health

## Abstract

There was a lack of evidence on the association between dairy intake and metabolic health status in overweight/obese adolescents. This study evaluated the association between dairy intake and metabolic health status in overweight/obese Iranian adolescents. Overweight/obese adolescents (n = 203; 101 boys and 102 girls) selected by a multistage cluster random sampling method have participated in this cross-sectional study. Dietary intake was assessed using a validated 147-item food frequency questionnaire. Anthropometric indices, blood pressure, fasting glucose, insulin, and lipid profile were measured. Participants were categorized to metabolically healthy obese (MHO) and metabolically unhealthy obese (MUO) according to International Diabetes Federation (IDF) criteria and a combination of IDF with Homeostasis Model Assessment Insulin Resistance (HOMA-IR) criteria. The frequency of MUO based on IDF, and IDF/HOMA-IR definitions was 38.9% and 33.0%, respectively. In fully-adjusted model, participants in the highest tertile of dairy intake had 61% lower odds of MUO based on IDF criteria (OR = 0.39, 95% CI 0.15–0.99). Higher dairy intake was associated with a non-significant lower risk of MUO according to IDF/HOMA-IR definition in the maximally-adjusted model (OR = 0.44, 95% CI 0.17–1.16). Stratifies analysis by sex and body mass index revealed that the association was stronger in girls and overweight subjects. Furthermore, higher intake of low-fat dairy was related to a reduced likelihood of MUO, while higher intake of high-fat dairy was related to increased odds of MUO. This community-based cross-sectional study revealed that higher intake of dairy was associated with a significant lower odd of MUO among Iranian adolescents, especially in girls and overweight subjects.

## Introduction

Childhood and adolescent overweight/obesity have been considered as one of the most serious public health challenges of the twenty-first century by the world health organization (WHO)^1^. The global prevalence of overweight/obesity in children and adolescents has increased tremendously since 1980^2^. Pooled results from population-based studies comprising 31.5 million individuals aged 5–19 years from 200 countries indicated that global age-standardized prevalence of obesity have increased in both sexes worldwide^3^. Overweight/obesity in adolescents imposes a substantial burden on affected individuals, their families, and health care systems, especially in low- and middle-income countries with limited resources. Excess body weight in adolescents often is associated with serious complications such as hypertension, dyslipidemia, insulin resistance, and fatty liver diseases which all convey an increased risk of future cardiovascular disease (CVD), type 2 diabetes mellitus, and subsequent premature death^4,5^. Thus, an effective management for overweight and obesity and their related complications in this age population is essential.

Despite the above-mentioned relations, cardiometabolic risk factors do not develop in some clinically obese individuals known as metabolically healthy obese (MHO) individuals^6^. There has been no consensus regarding the definition of MHO and different definitions and consequently, varying prevalence rates of this condition have been proposed in the literature^6,7^. However, it is evident that MHO individuals display a desirable metabolic profile presented by better insulin sensitivity, lipid profile, and blood pressure^8,9^ and lower mortality rate^10,11^. A combination of genetic and lifestyle-related factors such as dietary intake and physical activity plays a role in the etiology of this condition and prevention of developing metabolically unhealthy obese (MUO) condition^12,13^. For example, previous investigations have reported that healthy dietary patterns with higher intake of fruits and vegetables^14,15^ and lower intake of fats^12^ and soft drinks^13^ were related to MHO.

A limited number of previous studies have investigated the relationship between dairy intake and metabolic health status in adolescents; no consistency was seen between available results. A cohort study of 531 participants aged 6–18 years without the metabolic syndrome (MetS) at baseline showed a reduced risk for MetS incident among those with higher intake of dairy foods, mainly for low-fat milk and yogurt^16^. Abreu et al. have also showed that higher milk intake was associated with lower cardiometabolic risk score in adolescents. However, they found no association between total dairy, yogurt, or cheese intake and this score^17^. Conversely, the results of a cross-sectional study of 785 adolescents aged 10–19 years by Mohammadi et al. did not support the association between dairy intake and MetS or its components^18^. To the best of our knowledge, no previous study has addressed the association between dairy intake and MHO/MUO status in adolescents. The aim of the present study was to investigate the association between dairy intake and metabolic health status in Iranian overweight and obese adolescents.

## Methods

### Study design and participants

The current cross-sectional study was performed among a representative sample of Iranian adolescents. For sample size calculation, the prevalence of MUO was considered as 60%, based on previous studies among overweight and obese Iranian adolescents^19,20^. Considering a power 80%, type I error of 0.05, desired confidence interval (CI) of 0.95, and precision (d) of 7%, the required sample size was estimated to be 188. A stratified, multistage cluster sampling design was used to randomly select participants involving students aged 12–18 years old from 16 middle and high schools in six various education districts in Isfahan, Iran. The age-sex-specific percentile curves of body mass index (BMI)^21^ were used to screen overweight/obese adolescents and invite them to participate in the study. The exclusion criteria were having genetic or endocrine disorders such as type 1 diabetes mellitus, hypothyroidism, and Cushing’s syndrome, being on a weight-loss diet, taking nutritional supplements (including vitamin and mineral supplements) or medications that might influence metabolic markers such as body weight, lipid profile, blood glucose, or blood pressure. Finally, a total of 203 overweight/obese adolescents comprising 102 girls and 101 boys were included in the present study. The study protocol was approved by the ethics committee of the Isfahan University of Medical Sciences and a written informed consent was obtained from each participant and their parents.

### Assessment of dairy intakes

Dietary intakes of participants were assessed by a validated 147-item food frequency questionnaire (FFQ) completed by a trained nutritionist^22^. Previous investigations have documented that this questionnaire has a desirable validity and reliability for the assessment of food intake among Iranian adolescents^23,24^. The participants were asked to report the frequency of intake for each food item on a daily, weekly, and monthly basis. The amount of consumed food items was also reported based on standard portion sizes, and then the household measures were used to convert the portion sizes of consumed foods into grams per day^25^. Finally, Nutritionist IV software was applied to compute the daily intake of energy and nutrients. Dairy items in the applied FFQ were low-fat milk, whole milk, cocoa milk, regular yogurt, whole yogurt, strained yogurt, cheese, cream cheese, cream, ice cream, dough, and kashk. Sum of intakes of all dairy products was considered as total dairy intake. Low-fat dairy (< 2% fat) was considered as low-fat milk, regular yogurt, dough, and kashk. While high-fat dairy (> 2% fat) was considered as whole milk, cocoa milk, whole yogurt, strained yogurt, cheese, cream cheese, cream, and ice cream.

### Assessment of anthropometric indices and cardio-metabolic risk factors

Anthropometric indices were estimated by a trained nutritionist. Weight was measured by a digital scale (Seca Instruments, Germany) to the nearest 100 g while the participants were wearing thin clothes and no shoes. A stadiometer was used to measure height to the nearest 0.1 cm while the participants were standing with shoulders relaxed, and wearing no shoes. BMI was calculated by dividing weight (kg) by squared height (m^2^) and categorized according to the WHO growth curve of age-sex-specific BMI percentiles to normal weight (5th < BMI < 85th percentile), overweight (85th < BMI < 95th percentile), and obese (BMI > 95th percentile)^21^. Waist circumference (WC) was twice measured in midway between the lowest rib and the superior border of the iliac crest, after a normal expiration and without any pressure on the body surface, to the nearest 0.1 cm by an un-stretchable flexible anthropometric tape. The average of two measurements was considered as WC value. Blood pressure was measured twice after 15 min of rest on the right arm by a mercury sphygmomanometer in the morning and fasting condition.

Blood samples were collected after 12 h of fasting to assess biochemical indices. Fasting blood glucose (FBG) concentration was measured with an enzymatic colorimetric method using glucose oxidase (Pars Azmoon commercial kits, Tehran, Iran) on the day of blood collection. Serum insulin levels were measured using an ELISA kit (Diagnostic Biochem Canada Inc.). To estimate insulin resistance, the homeostasis model assessment insulin resistance (HOMA-IR) was calculated by the following formula: HOMA-IR = [(fasting insulin (mU/L) × FBG (mmol/L)]/22.5. Additionally, commercial kits (Pars Azmoon commercial kits, Tehran, Iran) were used to measure serum high-density lipoprotein cholesterol (HDL-c) and triglyceride (TG) levels.

### Assessment of metabolic health status

Two strategies were applied to demonstrate the metabolic health status (MHO vs. MUO): (1) based on the International Diabetes Federation (IDF) criteria, individuals with at least two of following risk factors were considered as MUO: increased TG (≥ 150 mg/dL), decreased HDL-c (< 40 mg/dL for the age of < 16 years, and < 50 mg/dL in girls/< 40 mg/dL in boys for the age of ≥ 16 years), increased fasting blood glucose (≥ 100 mg/dL) and increased blood pressure (≥ 130/85 mmHg)^26^, (2) based on a combination of the first strategy and the presence of insulin resistance (IR) based on HOMA-IR, MUO was considered as individuals with HOMA-IR > 3.16 and at least two mentioned metabolic risk factor; MHO was considered as subjects with HOMA-IR < 3.16, without considering the number of their cardiometabolic risk factors^27^.

### Assessment of other variables

Physical activity of participants was evaluated using the validated physical activity questionnaire for adolescents (PAQ-A) which consists of 9 items scoring from 1 to 5^28^. Eight items of this questionnaire are about usual activities such as their activity during spare time, physical education classes, lunch, after school, evenings, and weekends, as well as the frequency of physical activity during the last 7 days. The last item asks adolescents if they were prevented from engaging in regular physical activity by sickness or other reasons. Students were classified as sedentary (or not having an orderly week activity) (score < 2), low active (2 ≤ score < 3), moderately active (3 ≤ score < 4), and highly active (score ≥ 4), based on their total score of physical activity. A standard checklist was applied to collect the information regarding the students’ age, sex, and medical history of diseases, taking medications or supplements. A validated questionnaire was also used to gather information about socioeconomic variables (including parental job, parental education level, family size, having cars in the family, having computers/laptops, having a personal room, and taking trips in the last year)^29^.

### Statistical analysis

The normality of quantitative variables was examined using the Kolmogorov–Smirnov test. First, energy-adjusted dairy intake was calculated based on residual method^30^, in order to have an exposure of interest independent from energy intake. Continuous and categorical variables were respectively presented as mean ± SD/SE and frequency (percentage). Then, participants were categorized based on energy-adjusted tertiles of dairy intake. The chi-square test and one-way ANOVA were respectively used to determine categorical and continuous variables across tertiles of dairy intake. Additionally, energy, age, and sex-adjusted dietary intakes of subjects across tertiles of dairy intake were evaluated using analysis of covariance (ANCOVA). Odds ratios (ORs) and their attributed 95% confidence intervals (95% CIs) for MUO across tertiles of dairy intake were estimated by binary logistic regression in crude and multivariable-adjusted models. Based on previous literature^16,18,31^, in the first model, adjustments were made for age, sex, energy intake and fat intake (percent of energy). In the second model, physical activity and socioeconomic variables were additionally adjusted. Finally, BMI was added to the adjustments in the third model, to obtain an independent association from obesity. The first tertile of dairy intake was considered as the reference category in all models. To determine trends, tertiles of dairy intake were treated as an ordinal variable in logistic regression models. Furthermore, stratified analyses were performed based on BMI categories, sex, and low vs. high fat dairy intake. Data analyses were performed using SPSS version 20 software and the value of P < 0.05 was considered as statistically significant.

### Ethical approval and consent to participate

The study procedure was performed according to declaration of Helsinki and STROBE checklist. All participants provided informed written consent. The study protocol was approved by the local Ethics Committee of Isfahan University of Medical Sciences.

## Results

A total of 203 adolescents (101 boys and 102 girls) with a mean age of 13.98 ± 1.61 (SD) years and an average BMI of 27.35 ± 3.24 kg/m^2^ participated in the present study. Among participants, 38.9% (37 boys, and 42 girls) were classified as MUO based on IDF criteria, while 33% (35 boys, and 32 girls) were categorized as MUO based on IDF/HOMA-IR definition.

General characteristics and cardiometabolic factors of study participants across energy-adjusted tertiles of dairy intake are summarized in Table 1. Participants with the highest dairy intake (tertile 3) were more likely to be boys (P = 0.01), and physically active (P < 0.001) compared to those in the lowest tertile. Additionally, the mean FBG (P = 0.01) and HOMA-IR index (P = 0.03) were significantly lower among adolescents in the highest tertile in comparison to those in the lowest one. However, no significant difference was observed regarding other general or cardiometabolic features across tertiles of dairy intake. General characteristics and cardiometabolic factors of adolescents across energy-adjusted tertiles of dairy intake, stratified by sex are shown in the Supplemental Table 1. Both girls and boys in the highest tertile of dairy intake were more likely to be physically active. In addition, the mean FBG was significantly lower in subjects in the highest category of dairy intake in both genders (P < 0.05).

**Table 1:** General characteristics and cardiometabolic factors of study participants across energy-adjusted tertiles of dairy intake.

	Tertiles of dairy intake			
	T1 (n = 67) (< 430 g/day)	T2 (n = 68) (430–593 g/day)	T3 (n = 68) (> 593 g/day)	P-value^1^
**Sex, n (%)**				0.01
Boys	36 (53.7)	22 (32.4)	43 (63.2)	
Girls	31 (46.3)	46 (67.6)	25 (36.8)	
Age (year)	13.97 ± 1.51	14.15 ± 1.70	13.81 ± 1.62	0.47
Weight (kg)	75.16 ± 11.92	73.03 ± 11.31	72.27 ± 11.56	0.33
Height (cm)	164.61 ± 7.99	161.76 ± 6.70	164.54 ± 8.78	0.06
BMI (kg/m^2^)	27.61 ± 2.83	27.83 ± 3.46	26.63 ± 3.31	0.07
Waist circumference (cm)	91.27 ± 7.14	90.38 ± 9.15	89.35 ± 7.36	0.38
**Physical activity levels, n (%)**				< 0.001
Low	48 (71.6)	38 (50.0)	20 (29.4)	
High	19 (28.4)	34 (50.0)	48 (70.6)	
**Socioeconomic status**^**2**^**, n (%)**				0.47
Low	24 (35.8)	17 (25.0)	18 (26.5)	
Medium	30 (44.8)	30 (44.1)	30 (44.1)	
High	13 (19.4)	21 (30.9)	20 (29.4)	
Systolic blood pressure (mmHg)	115.09 ± 17.87	110.04 ± 21.11	113.01 ± 15.56	0.28
Diastolic blood pressure (mmHg)	74.32 ± 13.35	74.60 ± 6.80	71.57 ± 12.75	0.23
Fasting blood glucose (mg/dL)	100.75 ± 9.93	97.94 ± 7.46	95.75 ± 7.26	0.01
Insulin (μUI/mL)	23.53 ± 10.76	18.89 ± 14.42	18.90 ± 12.11	0.05
HOMA-IR index	5.88 ± 2.86	4.60 ± 3.50	4.59 ± 3.31	0.03
Triglycerides (mg/dL)	129.15 ± 62.94	129.18 ± 80.58	107.63 ± 51.45	0.09
HDL-c (mg/dL)	43.31 ± 8.40	44.63 ± 7.31	46.50 ± 7.82	0.06

Values are Mean ± SD; unless indicated.

*BMI* Body Mass Index, *HOMA* Homeostasis Model Assessment Insulin Resistance, *HDL-c* high-density lipoprotein cholesterol.

^1^Obtained from one-way ANOVA and χ^2^ test for quantitative and categorical variables, respectively.

^2^Socioeconomic status (SES) score was evaluated based on parental education level, parental job, family size, having car in the family, having computer/laptop, having personal room and having travel by using a validated questionnaire.

Dietary intakes of study participants across energy-adjusted tertiles of dairy intake, stratified by sex are presented in the Supplemental Table 2. In both groups, no significant difference was observed in energy intake across tertiles of dairy intake (P > 0.05). However, girls in the third tertile of dairy intake had a significantly higher intake of protein, cholesterol, saturated fatty acid (SFA), vitamin C, vitamin A, riboflavin, vitamin B6, folate, vitamin B12, magnesium, calcium, and total fiber (P < 0.05), while no significant difference was observed between dietary intake of poly-unsaturated fatty acid (PUFA), thiamin, niacin, and vitamin E across tertiles of dairy intake among girls (P > 0.05). Furthermore, boys in the third tertile of dairy intake had a significantly higher intake of protein, fat, cholesterol, SFA, monounsaturated fatty acid (MUFA), vitamin C, vitamin A, riboflavin, vitamin B6, folate, vitamin E, magnesium, calcium, total fiber and lower intake of carbohydrates, thiamin, and niacin (P < 0.05). No significant difference was observed between dietary intake of PUFA and vitamin B12 across tertiles of dairy intake among boys (P > 0.05).

**Figure 1:**
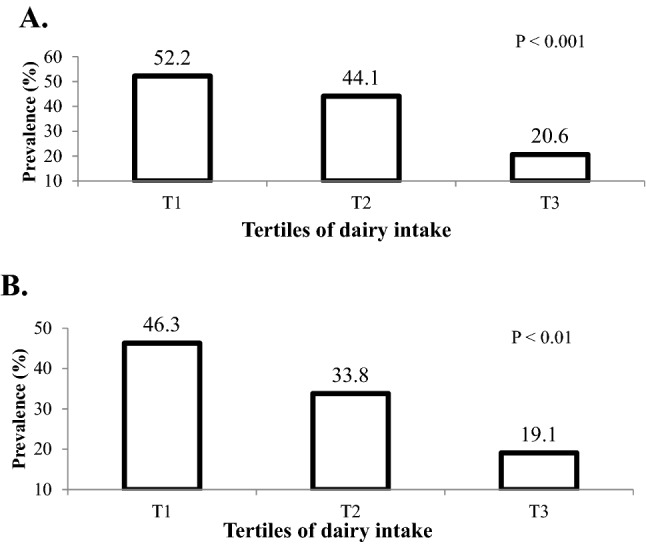
Prevalence of MUO based on IDF (**A**) and IDF/HOMA-IR definition (**B**) in energy-adjusted tertiles of dairy intake.

The distribution of adolescents with MUO phenotype across energy-adjusted tertiles of dairy intake is presented in Fig. 1. Based on IDF definition, 52.2%, 44.1%, and 20.6% of individuals were identified as MUO in tertiles 1, 2 and 3 of dairy intake (P < 0.001). In addition, based on IDF/HOMA-IR definition, the frequency of adolescents with MUO phenotype across tertiles of dairy intake was respectively 46.3%, 33.8%, and 19.1% (P < 0.001).

Multivariate adjusted odds ratio (OR) and 95% confidence interval (CI) for MUO across energy-adjusted tertiles of dairy intake are indicated in Table 2. Based on IDF criteria, individuals in the highest tertile of dairy intake had 76% lower odds of MUO, compared to those in the bottom tertile, in the crude model (OR: 0.24, 95% CI 0.11–0.51). This association remained significant after adjustment for potential confounders. Such that, in fully-adjusted model, adolescents in the highest tertile had 61% lower odds of MUO in comparison to the reference tertile (OR: 0.39, 95% CI 0.15–0.99). A significant inverse association was also observed between energy-adjusted dairy intake and odds of MUO based on IDF/HOMA-IR criteria in the crude model (OR: 0.27, 95% CI 0.13–0.59). Nevertheless, when physical activity and socioeconomic status were taken into account as the confounders, this association disappeared. So, dairy intake was associated with a non-significant decreased odd of MUO, in the fully-adjusted model (OR: 0.44, 95% CI 0.17–1.16).

**Table 2:** Multivariate adjusted odds ratio (OR) and 95% confidence interval (CI) for MUO across energy-adjusted tertiles of dairy intake.

	Tertiles of dairy intake			
	T1 (n = 67) (< 430 g/day)	T2 (n = 68) (430–593 g/day)	T3 (n = 68) (> 593 g/day)	P-trend
**MUO phenotype based on IDF criteria**				
Cases (n)	35	30	14	
Crude	1	0.72 (0.37–1.42)	0.24 (0.11–0.51)	< 0.001
Model 1^2^	1	0.80 (0.39–1.65)	0.21 (0.09–0.49)	< 0.001
Model 2^3^	1	1.27 (0.56–2.89)	0.39 (0.15–0.98)	0.08
Model 3^4^	1	1.27 (0.55–2.91)	0.39 (0.15–0.99)	0.08
**MUO phenotype based on IDF/HOMA-IR criteria**				
Cases (n)	31	23	13	
Crude	1	0.59 (0.30–1.19)	0.27 (0.13–0.59)	0.01
Model 1^2^	1	0.72 (0.34–1.52)	0.24 (0.10–0.56)	0.01
Model 2^3^	1	1.00 (0.43–2.33)	0.44 (0.17–1.15)	0.12
Model 3^4^	1	0.98 (0.42–2.32)	0.44 (0.17–1.16)	0.13

^1^All values are odds ratios and 95% confidence intervals.

^2^Model 1: Adjusted for age, sex, total energy intake, and fat intake (percent of energy).

^3^Model 2: Additionally, adjusted for physical activity and socioeconomic status (parental education, parental job, number of family members, having car in the family, having computer/laptop, having personal room and having trip).

^4^Model 3: Additionally adjusted for body mass index (BMI).

Multivariate adjusted odds ratio (OR) and 95% confidence interval (CI) for MUO across energy-adjusted tertiles of dairy intake, stratified by BMI categories are shown in Table 3. According to IDF definition, the highest category of dairy intake was significantly associated with 88% decreased odds of MUO both in the crude (OR: 0.12, 95% CI 0.03–0.41) and fully adjusted model (OR: 0.12, 95% CI 0.02–0.66), among overweight adolescents. On the other hand, among obese adolescents, no significant association was observed between dairy intake and MUO in the crude (OR: 0.44, 95% CI 0.16–1.23) or fully adjusted model (OR: 0.66, 95% CI 0.19–2.21), based on IDF definition. The same pattern was observed for the association between dairy intake and MUO among overweight and obese adolescents, based on IDF/HOMA-IR criteria. Such that, overweight individuals in the highest tertile of dairy intake had 89% and 88% reduced odds of MUO in the crude (OR: 0.11, 95% CI 0.03–0.44) and fully-adjusted model (OR: 0.12, 95% CI 0.02–0.73). However, there was no significant association between dairy intake and MUO either in the crude (OR: 0.56, 95% CI 0.20–1.53) and fully-adjusted model (OR: 0.86, 95% CI 0.25–2.90), among obese adolescents.

**Table 3:** Multivariate adjusted odds ratio (OR) and 95% confidence interval (CI) for MUO across energy-adjusted tertiles of dairy intake, stratified by BMI categories^1^.

	Tertiles of dairy intake			
	T1 (n = 67) (< 430 g/day)	T2 (n = 68) (430–593 g/day)	T3 (n = 68) (> 593 g/day)	P-trend
**Overweight**				
(Cases/participants)	15/31	9/33	4/40	
Crude	1	0.40 (0.14–1.13)	0.12 (0.03–0.41)	0.01
Model 1^1^	1	0.37 (0.11–1.18)	0.09 (0.02–0.36)	0.01
Model 2^2^	1	0.78 (0.18–3.34)	0.12 (0.02–0.66)	0.02
**Obese**				
(Cases/participants)	20/36	21/35	10/28	
Crude	1	1.20 (0.47–3.08)	0.44 (0.16–1.23)	0.14
Model 1^1^	1	1.33 (0.49–3.37)	0.39 (0.13–1.17)	0.14
Model 2^2^	1	1.73 (0.58–5.17)	0.66 (0.19–2.21)	0.69
**Overweight**				
(Cases/participants)	13/31	4/33	3/40	
Crude	1	0.19 (0.05–0.68)	0.11 (0.03–0.44)	
Model 1^1^	1	0.22 (0.06–0.86)	0.08 (0.02–0.39)	0.01
Model 2^2^	1	0.37 (0.07–1.98)	0.12 (0.02–0.73)	0.01
**Obese**				0.02
(Cases/participants)	18/36	19/35	10/28	
Crude	1	1.19 (0.47–3.02)	0.56 (0.20–1.53)	0.29
Model 1^1^	1	1.33 (0.50–3.55)	0.49 (0.16–1.49)	0.29
Model 2^2^	1	1.63 (0.56–4.78)	0.86 (0.25–2.90)	0.99

All values are odds ratios and 95% confidence intervals.

^1^Model 1: Adjusted for age, sex, total energy intake, and fat intake (percent of energy).

^2^Model 2: Additionally adjusted for physical activity and socioeconomic status (parental education, parental job, number of family members, having car in the family, having computer/laptop, having personal room and having trip).

Multivariate adjusted odds ratio (OR) and 95% confidence interval (CI) for MUO across energy-adjusted tertiles of dairy intake, stratified by sex are presented in Table 4. In the crude model, girls in the top category of dairy intake had 90% and 91% reduced odds of MUO phenotype according to IDF (OR: 0.10, 95% CI 0.02–0.40) and IDF-HOMA-IR definition (OR: 0.09, 95% CI 0.02–0.46), compared to girls in the bottom category. These relationships remained significant after adjustment for potential confounders. Such that, in fully adjusted model, girls in the third tertile of dairy intake were 89% (OR: 0.11, 95% CI 0.02–0.58) and 92% (OR: 0.08, 95% CI 0.01–0.61) less likely to have MUO phenotype based on IDF and IDF/HOMA-IR definitions, respectively. Among boys, a significant inverse association was observed between dairy intake and MUO based on IDF definition in the crude model (OR: 0.38, 95% CI 0.15–0.99). The association disappeared after adjustment for potential confounders (OR: 0.80, 95% CI 0.22–2.89). No significant association was observed between dairy intake and MUO based on IDF/HOMA-IR definition either in the crude (OR: 0.43, 95% CI 0.17–1.11) and fully-adjusted model (OR: 1.01, 95% CI 0.28–3.72) among boys.

**Table 4:** Multivariate adjusted odds ratio (OR) and 95% confidence interval (CI) for MUO across energy-adjusted tertiles of dairy intake, stratified by sex.

	Tertiles of dairy intake			
	T1 (n = 67) (< 430 g/day)	T2 (n = 68) (430–593 g/day)	T3 (n = 68) (> 593 g/day)	P-trend
**MUO phenotype based on IDF criteria**				
Girl				
(Cases/participants)	18/31	21/46	3/25	
Crude	1	0.61 (0.24–1.52)	0.10 (0.02–0.40)	0.01
Model 1^1^	1	0.71 (0.26–1.92)	0.08 (0.02–0.38)	0.01
Model 2^2^	1	1.06 (0.34–3.37)	0.11 (0.02–0.58)	0.02
Model 3^3^	1	1.07 (0.34–3.38)	0.11 (0.02–0.58)	0.02
Boy				
(Cases/participants)	17/36	9/22	11/43	
Crude	1	0.77 (0.27–2.26)	0.38 (0.15–0.99)	0.04
Model 1	1	0.86 (0.28–2.61)	0.36 (0.12–1.06)	0.07
Model 2	1	1.27 (0.35–4.59)	0.68 (0.19–2.45)	0.61
Model 3	1	1.39 (0.36–5.30)	0.80 (0.22–2.89)	0.79
**MUO phenotype based on IDF/HOMA-IR criteria**				
Girl				
(Cases/participants)	15/31	15/46	2/25	
Crude	1	0.52 (0.20–1.32)	0.09 (0.02–0.46)	0.01
Model 1	1	0.62 (0.22–1.75)	0.07 (0.01–0.43)	0.01
Model 2	1	0.77 (0.23–2.53)	0.09 (0.01–0.67)	0.03
Model 3	1	0.71 (0.21–2.36)	0.08 (0.01–0.61)	0.02
Boy				
(Cases/participants)	16/36	8/22	11/43	
Crude	1	0.17 (0.24–2.12)	0.43 (0.17–1.11)	0.08
Model 1	1	0.83 (0.27–2.59)	0.44 (0.15–1.29)	0.14
Model 2	1	1.26 (0.34–4.67)	0.87 (0.24–3.17)	0.87
Model 3	1	1.33 (0.34–5.18)	1.01 (0.28–3.72)	0.94

All values are odds ratios and 95% confidence intervals.

^1^Model 1: Adjusted for age, sex, total energy intake, and fat intake (percent of energy).

^2^Model 2: Additionally adjusted for physical activity and socioeconomic status (parental education, parental job, number of family members, having car in the family, having computer/laptop, having personal room and having trip).

^3^Model 3: Additionally adjusted for body mass index (BMI).

As reported in Table 5, we have also evaluated the association between MUO phenotype with high- and low-fat dairy intake. In the crude model, individuals in the top tertile of low-fat dairy intake had 81% (OR: 0.19, 95% CI 0.09–0.40) and 84% (OR: 0.16, 95% CI 0.07–0.36) lower odds of MUO based on IDF and IDF/HOMA-IR definitions, respectively. These associations remained significant after adjustment for confounding variables. Such that, in fully-adjusted model, adolescents in the third tertile of low-fat dairy intake compared to the first tertile had 64% (OR: 0.36, 95% CI 0.14–0.89) and 75% (OR: 0.25, 95% CI 0.09–0.63) reduced likelihood of MUO, based on IDF and IDF/HOMA-IR definitions, respectively. No significant association was found between high-fat dairy intake and MUO phenotype based on IDF criteria in the crude (OR: 1.91, 95% CI 0.96–3.83) and fully adjusted (OR: 1.62, 95% CI 0.67–3.93) models. While, a significant positive association was observed between high-fat dairy intake and MUO based on IDF/HOMA-IR in the crude model (OR: 2.61, 95% CI 1.26–5.41). This association was strengthened after adjustment for confounding variables (OR: 3.00, 95% CI 1.16–7.76).

**Table 5:** Multivariate adjusted odds ratio (OR) and 95% confidence interval (CI) for MUO across energy-adjusted tertiles of low- and high-fat dairy intake.

	Tertiles of low-fat dairy				Tertiles of high-fat dairy			
	T1 (n = 67) (< 174 g/day)	T2 (n = 68) (174–947 g/day)	T3 (n = 68) (> 947 g/day)	P-trend	T1 (n = 67) (< 57 g/day)	T2 (n = 68) (57–280 g/day)	T3 (n = 68) (> 280 g/day)	P-trend
**MUO phenotype based on IDF criteria**								
Cases (n)	39	26	14		23	22	34	
Crude	1	0.44 (0.22–0.88)	0.19 (0.09–0.40)	< 0.001	1	0.92 (0.45–1.87)	1.91 (0.96–3.83)	0.07
Model 1^1^	1	0.42 (0.20–0.86)	0.18 (0.08–0.40)	< 0.001	1	1.18 (0.55–2.54)	2.30 (1.07–4.94)	0.07
Model 2^2^	1	0.59 (0.26–1.31)	0.35 (0.14–0.86)	0.07	1	1.08 (0.46–2.57)	1.69 (0.70–4.06)	0.44
Model 3^3^	1	0.59 (0.26–1.31)	0.36 (0.14–0.89)	0.08	1	1.10 (0.46–2.63)	1.62 (0.67–3.93)	0.52
**MUO phenotype based on HOMA-IR criteria**								
Cases (n)	35	22	10		17	18	32	
Crude	1	0.44 (0.22–0.88)	0.16 (0.07–0.36)	< 0.001	1	1.06 (0.49–2.29)	2.61 (1.26–5.41)	0.01
Model 1^1^	1	0.41 (0.20–0.87)	0.14 (0.06–0.34)	< 0.001	1	1.63 (0.70–3.77)	3.74 (1.64–8.52)	0.01
Model 2^2^	1	0.56 (0.25–1.28)	0.24 (0.09–0.65)	0.02	1	1.74 (0.67–4.46)	3.24 (1.27–8.29)	0.05
Model 3^3^	1	0.56 (0.25–1.28)	0.25 (0.09–0.69)	0.03	1	1.79 (0.69–4.64)	3.00 (1.16–7.76)	0.07

All values are odds ratios and 95% confidence intervals.

^1^Model 1: Adjusted for age, sex, total energy intake, and fat intake (percent of energy).

^2^Model 2: Additionally, adjusted for physical activity and socioeconomic status (parental education, parental job, number of family members, having car in the family, having computer/laptop, having personal room and having trip).

^3^Model 3: Additionally adjusted for body mass index (BMI).

## Discussion

The present study indicated that more than 30% of Iranian adolescents have MUO phenotype, based on IDF or IDF/HOMA-IR definitions. Additionally, higher dairy intake in adolescents was significantly associated with reduced odds of MUO, based on both definitions. This association was stronger among girls and overweight individuals. However, the relationship between dairy intake and MUO among boys and obese subjects was dependent to covariates. Our findings also indicated that there was a significant inverse association between low-fat dairy intake and MUO according to both IDF, and IDF/HOMA-IR definitions, even after considering potential confounders, while a significant positive association was found between higher intake of high-fat dairy and MUO based on IDF/HOMA-IR definition. To the best of our knowledge, this was the first study that investigated the association between dairy intake and metabolic health status in overweight and obese adolescents.

In the present study, the comparison of metabolic health status components between tertiles of dairy intake showed that blood pressure values (SBP and DBP) had no significant difference across tertiles, while others factors including TG, HDL-c, and insulin levels had marginally significant differences across tertiles. In case of FBS and HOMA-IR, significant differences were observed between tertiles of dairy intake. These findings suggested that the metabolically unhealthy overweight/obese in adolescents could be regarded as more than the accumulation of the predisposing effects of its individual components.

Although the MHO phenotype is not essentially related to a better clinical outcome, prevention from shifting to MUO or even maintaining MHO status from adolescence to adulthood is valuable^32,33^. Our findings have suggested that overweight or obese adolescents can be clinically advised to increase their dairy intake, especially low-fat dairy intake, as an efficient strategy for achieving these goals.

Few previous studies have evaluated the association between dairy intake and metabolic syndrome (MetS) among children and adolescents with inconclusive results. None of these studies have reported their results across sex and BMI categories. Tehran Lipid and Glucose Study, a prospective investigation with 6.6 years follow-up on 531 healthy adolescents aged 6–18 years showed that higher dairy consumption was associated with reduced risk of MetS and some components of MetS, such as abdominal obesity, hypertension, and high serum TG levels^16^. Similar to our results, higher consumption of low-fat milk, and yogurt as well as moderate consumption of regular cheese in the mentioned cohort study was associated with reduced risk of MetS. While no significant relationship was observed between high-fat dairy intake and risk of MetS incidence^16^. Abreu et al. in a cross-sectional study examined the relationship between dairy intake and cardiometabolic risk score in adolescents aged 15 to 18 years. The cardiometabolic risk score was computed by summing up the age- and sex-adjusted z scores of total cholesterol/HDL-c, TG, HOMA, body fat percentage, systolic blood pressure, and cardiorespiratory fitness^17^. The results of this study showed that only a higher intake of milk was associated with reduced odds of cardiometabolic risk score. While no association was observed between cardiometabolic risk score and higher intake of total dairy, yogurt, and cheese^17^. In contrast to the findings of the above-mentioned studies, the study by Mohammadi et al. on 785 Iranian adolescents aged 10–19 years showed that there was no significant relationship between total, low-fat and high-fat dairy as well as specific dairy products such as milk, yogurt, and cheese and odds of MetS^18^. The observed inconsistencies in the findings of these studies are probably due to differences in study design, studied population, and potential confounders considered in the analyses.

Several potential mechanisms have been suggested explaining the relation of dairy intake and metabolic health status. It has been hypothesized that calcium, which is abundant in dairy products, interferes with fat absorption in the intestine via binding to saturated fatty acids and forming insoluble soaps; thereby may improve serum TG levels and HDL-c to LDL-c ratio^34,35^. Dietary calcium intake from dairy also affects calciotropic hormones and reduces intracellular calcium which inhibits the synthesis of fatty acids, and induces lipolysis^36,37^. In addition, bioactive peptides of fermented dairy products such as yogurt and cheese have beneficial influences on blood pressure and fat accumulation through inhibiting angiotensin-I converting enzyme^38,39^. Some previous researches have also proposed gut microbiota dysbiosis in overweight/obese children and adolescents^40,41^. Fermented dairy products that contain probiotic bacteria can possibly improve the metabolic status of overweight/obese adolescents by various pathways including modulating insulin sensitivity, inflammatory reactions, and fat metabolism^42,43^.

The present study has several limitations that should be addressed. First of all, cross-sectional design of this study did not allow us to establish a causal relationship between dairy intake and metabolic health status. In addition, the possibility of reverse causality should be considered in the study, since overweight/obese adolescents with MUO might change their dietary intakes to attenuate their symptoms. Furthermore, our sample of overweight and obese adolescents was relatively small and thus it was impossible to stratify the analysis based on each dairy product. We also did not gather data of other risk factors that might affect metabolic health status such as pubertal status, paternal obesity, the time of adiposity rebound, and sleep behaviors. Finally, self-reported dietary intakes could be biased, in spite of using a validated FFQ. However, the current study was conducted on a somehow representative sample of Iranian overweight/obese adolescents, although our sample was not large. The preliminary results of the study can be used for designing future prospective or clinical trial investigations.

In conclusion, the findings of the present cross-sectional study indicated that a higher intake of dairy was associated with lower odds of MUO among Iranian adolescents, especially in girls and overweight subjects. Further large-scale prospective studies are needed to confirm these results.

## Supplementary Information

Supplementary Tables.

## Data Availability

The data that support the findings of this study are available from the corresponding author upon reasonable request.

## Abbreviations

FFQ: Food frequency questionnaire
OR: Odds ratios
95% CI: 95% Confidence interval
BMI: Body mass index
MHO: Metabolically healthy obese
MUO: Metabolically unhealthy obese
IDF: International Diabetes Federation
HOMA-IR: Homeostasis Model Assessment Insulin Resistance
IR: Insulin resistance
PAQ-A: Physical Activity Questionnaire for Adolescents
SES: Socioeconomic status
SFA: Saturated fatty acids
WHO: World Health Organization
WC: Waist circumference
BP: Blood pressure
SBP: Systolic blood pressure
DBP: Diastolic blood pressure
TG: Triglycerides
HDL-c: High density lipoprotein cholesterol
FBG: Fasting blood glucose
ANCOVA: Analysis of covariance
SPSS: Statistical package for the social sciences
SD: Standard deviation
SE: Standard error
