# Supplementary material for: Dairy intake in relation to metabolic health status in overweight and obese adolescents
Source: Sci Rep. 2022 Nov 1;12:18365. doi: 10.1038/s41598-022-22827-4 (PMC9626638; doi:10.1038/s41598-022-22827-4)
Supplement: Supplementary file 1 — Supplementary Tables. [file 41598_2022_22827_MOESM1_ESM.docx]

| **Supplemental Table 1. General characteristics and cardiometabolic factors of study participants across energy-adjusted** **tertiles of dairy intake, stratified by sex^1^** | | | | | | | | |
| --- | --- | --- | --- | --- | --- | --- | --- | --- |
|  | Girls (n=102) | | | | Boys (n=101) | | | |
|  | Tertiles of dairy intake | | | | Tertiles of dairy intake | | | |
|  | T1  (n=31)  (<430 g/d) | T2  (n=46)  (430-593 gr/d) | T3  (n=25)  (>593 g/d) | P-value^2^ | T1  (n=36)  (<430 g/d) | T2  (n=22)  (430-593 gr/d) | T3  (n=43)  (>593 g/d) | P-value^2^ |
| Age (year) | 14.55$\pm$1.39 | 14.63$\pm$1.62 | 14.64$\pm$1.75 | 0.97 | 13.47$\pm$1.44 | 13.14$\pm$1.39 | 13.33$\pm$1.34 | 0.67 |
| Weight (kg) | 74.05$\pm$10.39 | 73.05$\pm$10.22 | 70.48$\pm$11.11 | 0.43 | 76.13$\pm$13.16 | 72.98$\pm$13.57 | 73.31$\pm$11.81 | 0.54 |
| Height (cm) | 161.84$\pm$5.33 | 160.46$\pm$5.46 | 160.75$\pm$6.35 | 0.56 | 166.99$\pm$9.13 | 164.47$\pm$8.25 | 166.75$\pm$9.30 | 0.55 |
| BMI (kg/m^2^) | 28.19$\pm$2.89 | 28.33$\pm$3.38 | 27.29$\pm$4.30 | 0.47 | 27.11$\pm$2.73 | 26.80$\pm$3.47 | 26.24$\pm$2.55 | 0.39 |
| Waist circumference (cm) | 90.65$\pm$6.56 | 89.92$\pm$7.49 | 87.94$\pm$6.52 | 0.34 | 91.80$\pm$7.66 | 91.34$\pm$12.06 | 90.17$\pm$7.75 | 0.70 |
| Physical activity levels, n (%) |  |  |  | <0.001 |  |  |  | 0.02 |
| Low | 87.1 | 56.5 | 32.0 |  | 58.3 | 36.4 | 27.9 |  |
| High | 12.9 | 43.5 | 68.0 |  | 41.7 | 63.6 | 72.1 |  |
| Socioeconomic status^3^, n (%) |  |  |  | 0.28 |  |  |  | 0.71 |
| Low | 45.2 | 26.1 | 32.0 |  | 27.8 | 22.7 | 23.3 |  |
| Medium | 45.2 | 50.0 | 56.0 |  | 44.4 | 31.8 | 37.2 |  |
| High | 9.7 | 23.9 | 12.0 |  | 27.8 | 45.5 | 39.5 |  |
| Systolic blood pressure (mmHg) | 110.48$\pm$22.74 | 106.07$\pm$23.49 | 108.64$\pm$22.54 | 0.70 | 119.06$\pm$11.12 | 118.36$\pm$11.51 | 115.56$\pm$8.83 | 0.29 |
| Diastolic blood pressure (mmHg) | 73.90$\pm$14.20 | 74.33$\pm$6.93 | 67.72$\pm$19.37 | 0.11 | 74.68$\pm$12.76 | 75.18$\pm$6.66 | 73.81$\pm$5.57 | 0.83 |
| Fasting blood glucose (mg/dL) | 99.26$\pm$10.55 | 97.74$\pm$7.82 | 93.08$\pm$5.21 | 0.02 | 102.03$\pm$9.32 | 98.36$\pm$6.79 | 97.30$\pm$7.87 | 0.04 |
| Insulin (μUI/mL) | 21.13$\pm$10.12 | 18.80$\pm$16.46 | 13.54$\pm$5.49 | 0.08 | 25.59$\pm$11.00 | 19.07$\pm$9.13 | 22.01$\pm$13.77 | 0.12 |
| HOMA-IR index | 5.23$\pm$2.73 | 4.57$\pm$3.96 | 3.16$\pm$1.42 | 0.05 | 6.44$\pm$2.90 | 4.67$\pm$2.34 | 5.42$\pm$3.79 | 0.11 |
| Triglycerides (mg/dL) | 121.19$\pm$55.62 | 115.30$\pm$75.26 | 75.26$\pm$47.88 | 0.23 | 136.00$\pm$68.67 | 158.18$\pm$85.30 | 116.21$\pm$52.04 | 0.05 |
| HDL-c (mg/dL) | 43.39$\pm$7.94 | 45.46$\pm$7.07 | 47.48$\pm$5.99 | 0.11 | 43.25$\pm$8.88 | 42.91$\pm$7.68 | 45.93$\pm$8.72 | 0.27 |
| 1 Values are Mean±SD; unless indicated. Abbreviations: BMI: Body Mass Index; HOMA: Homeostasis Model Assessment Insulin Resistance; HDL-c: high-density lipoprotein cholesterol.  2 Obtained from one-way ANOVA and χ2 test for quantitative and categorical variables, respectively.  3 Socioeconomic status (SES) score was evaluated based on parental education level, parental job, family size, having car in the family, having computer/laptop, having personal room and having travel by using a validated questionnaire. | | | | | | | | |

| **Supplemental Table 2. Dietary intakes (energy and macro/micro nutrients) of study participants across energy-adjusted tertiles of dairy intake, stratified by sex^1^** | | | | | | | | |
| --- | --- | --- | --- | --- | --- | --- | --- | --- |
|  | Girls (n=102) | | | | Boys (n=101) | | | |
|  | Tertiles of dairy intake | | | | Tertiles of dairy intake | | | |
|  | T1  (n=31)  (<430 g/d) | T2  (n=46)  (430-593 gr/d) | T3  (n=25)  (>593 g/d) | P-value^2^ | T1  (n=36)  (<430 g/d) | T2  (n=22)  (430-593 gr/d) | T3  (n=43)  (>593 g/d) | P-value^2^ |
| Energy, kcal | 2652.78$\pm$54.92 | 2555.36$\pm$45.08 | 2539.76$\pm$61.15 | 0.29 | 3193.59$\pm$116.29 | 3121.87$\pm$148.84 | 3216.89$\pm$106.22 | 0.87 |
| Protein, % of energy | 12.97$\pm$0.34 | 14.43$\pm$0.28 | 15.78$\pm$0.38 | <0.001 | 13.09$\pm$0.27 | 14.12$\pm$0.34 | 15.40$\pm$0.25 | <0.001 |
| Carbohydrate, % of energy | 59.99$\pm$0.89 | 58.17$\pm$0.73 | 57.31$\pm$0.99 | 0.11 | 61.30$\pm$0.75 | 59.26$\pm$0.96 | 54.75$\pm$0.69 | <0.001 |
| Fat, % of energy | 28.39$\pm$0.92 | 28.82$\pm$0.76 | 28.67$\pm$1.03 | 0.93 | 26.73$\pm$0.81 | 28.11$\pm$1.04 | 31.46$\pm$0.74 | <0.001 |
| Cholesterol, mg | 209.66$\pm$12.86 | 253.10$\pm$10.48 | 260.81$\pm$14.23 | 0.01 | 266.28$\pm$17.93 | 309.69$\pm$27.98 | 376.72$\pm$16.39 | <0.001 |
| SFA, gr | 21.79$\pm$0.90 | 24.91$\pm$0.73 | 25.72$\pm$0.99 | 0.007 | 25.90$\pm$0.81 | 29.88$\pm$1.04 | 34.85$\pm$0.74 | <0.001 |
| MUFA, gr | 22.61$\pm$1.03 | 24.42$\pm$0.84 | 25.02$\pm$1.14 | 0.24 | 28.28$\pm$1.21 | 28.99$\pm$1.55 | 34.58$\pm$1.11 | <0.001 |
| PUFA, gr | 28.66$\pm$1.30 | 25.17$\pm$1.06 | 23.58$\pm$1.44 | 0.03 | 30.13$\pm$1.44 | 31.50$\pm$1.84 | 31.84$\pm$1.31 | 0.67 |
| Vitamin C, mg | 105.05$\pm$9.37 | 117.86$\pm$7.64 | 146.80$\pm$10.36 | 0.01 | 118.64$\pm$9.63 | 160.97$\pm$12.33 | 162.01$\pm$8.80 | 0.002 |
| Vitamin A, RAE | 775.30$\pm$116.89 | 942.98$\pm$95.28 | 1195.12.32$\pm$129.33 | 0.06 | 1088.86$\pm$9.63 | 1125.81$\pm$122.02 | 1477.93$\pm$87.02 | 0.006 |
| Thiamin, mg | 2.51$\pm$0.05 | 2.39$\pm$0.04 | 2.30$\pm$0.05 | 0.02 | 3.10$\pm$0.05 | 2.93$\pm$0.07 | 2.69$\pm$0.05 | <0.001 |
| Riboflavin, mg | 1.55$\pm$0.05 | 2.05$\pm$0.04 | 2.47$\pm$0.05 | <0.001 | 1.93$\pm$0.06 | 2.53$\pm$0.07 | 3.21$\pm$0.05 | <0.001 |
| Niacin, mg | 26.10$\pm$0.50 | 24.85$\pm$0.41 | 23.08$\pm$0.55 | <0.001 | 32.88$\pm$0.55 | 30.93$\pm$0.71 | 27.98$\pm$0.51 | <0.001 |
| Vitamin B6, mg | 1.32$\pm$0.06 | 1.44$\pm$0.05 | 1.60$\pm$0.07 | 0.01 | 1.55$\pm$0.07 | 1.91$\pm$0.09 | 1.96$\pm$0.06 | <0.001 |
| Vitamin E, mg | 32.81$\pm$1.85 | 26.48$\pm$1.51 | 23.97$\pm$2.05 | <0.001 | 32.33$\pm$2.05 | 32.62$\pm$2.63 | 33.65$\pm$1.88 | <0.001 |
| Folate, mcg | 237.15$\pm$14.54 | 291.07$\pm$11.85 | 350.22$\pm$16.09 | <0.001 | 272.60$\pm$15.12 | 355.62$\pm$19.37 | 398.63$\pm$13.81 | <0.001 |
| Vitamin B12, mcg | 2.75$\pm$0.17 | 3.95$\pm$0.14 | 4.82$\pm$0.19 | 0.005 | 3.70$\pm$0.20 | 4.71$\pm$0.26 | 6.44$\pm$0.19 | 0.88 |
| Magnesium, mg | 216.47$\pm$7.68 | 261.21$\pm$6.26 | 299.16$\pm$8.49 | <0.001 | 260.45$\pm$8.66 | 321.75$\pm$11.10 | 368.41$\pm$7.91 | <0.001 |
| Calcium, mg | 869.86$\pm$28.98 | 1188.38$\pm$23.62 | 1518.82$\pm$32.06 | <0.001 | 1102.46$\pm$33.83 | 1474.87$\pm$43.34 | 1854.85$\pm$30.91 | <0.001 |
| Total fiber, gr | 16.72$\pm$0.79 | 17.18$\pm$0.65 | 19.59$\pm$0.88 | 0.04 | 19.58$\pm$0.80 | 22.30$\pm$1.02 | 22.19$\pm$0.73 | 0.03 |
| 1 Values are Mean±SE. Energy intake and macronutrients were adjusted for age and sex; all other values were adjusted for age, sex and energy intake.  2 P-value obtained from ANCOVA test for adjustment of energy intake.  Abbreviations: SFA, Saturated fatty acids; MUFA, Monounsaturated fatty acids; PUFA, Polyunsaturated fatty acids. | | | | | | | | |
